# Surgical resection for gastrointestinal stromal tumors (GIST): experience on 25 patients

**DOI:** 10.1186/1477-7819-3-78

**Published:** 2005-12-30

**Authors:** Luigi Boni, Angelo Benevento, Gianlorenzo Dionigi, Francesca Rovera, Renzo Dionigi

**Affiliations:** 1 Department of Surgery, University of Insubria, Ospedale di Circolo e Fondazione Macchi, Varese, Italy

## Abstract

**Background:**

Gastrointestinal stromal tumors (GIST) are infrequent and diagnosis and prognosis could be troublesome. We present short and long term results of surgical resection for GIST at the Department of Surgery, University of Insubria, during a period of 17 years.

**Materials and methods:**

All patients' data, tumor characteristics, surgical procedure and survival data were analyzed retrospectively. Tumors were divided in risk classes using the classification proposed by Fletcher, based on tumor size and number of mitosis.

**Results:**

Between 1987 and 2004, 25 patients underwent surgical resection for GIST. Stomach was the most common site of localization. Complete resection was achieved in 88% cases, while in 12% radical resection was not possible. The mean tumor size was 9.2 cm (1.2 – 30 cm): <5 cm diameter in 14/25 cases (56%), 5–10 cm in 5/25 (20%) and >10 cm in 6/25 (24%). Mitotic count was <10/50 HPF in 68% (17/25) and >10/50 in 32% (8/25). Using Fletcher's classification, tumors were divided in very low (11/25, 44%), low (4/25, 16%), intermediate (6/25, 24%) and high-risk (4/25, 16%) groups. The 5-year overall survival was 65% and 34% respectively with a statistically significant difference between tumors <5 cm and >10 cm in diameter and between complete and incomplete resection. High-risk tumors had a significantly shorter survival than low or very low risk.

**Conclusion:**

Our experience confirms that GIST's are uncommon and aggressive cancers. The prognosis is strictly related to tumor size and number of mitosis. Although significant advances on new chemotherapeutic regimes have been made, to date, only radical surgery offers the chance of long-term survival.

## Background

Gastrointestinal stromal tumors (GIST) are extremely unusual neoplasm accounting for less than 1% of all gastrointestinal tumors, arising from the Cajal's interstitial cells located in mesodermal tissue. They are defined as primary mesenchymal tumors, typically staining positive for the expression of c-KIT protein [1]. Diagnosis is complex and always requires immunohistochemical staining, since it is based on specific ultrastructural characteristics and positivity for specific immunophenotype markers [1,2].

Overall survival after surgical resection and clinical behavior of GIST are depending to tumor size and mitotic count, regardless their benign or malignant microscopic features [3]. Fletcher *et al *[2] divided GIST in different classes of risk, based on the analysis of prognostic factors [4,5] classifying these tumors in different "risk classes".

Despite recent advances in chemotherapeutic regimens, such as introduction of targeted therapy with inhibitors of tyrosine kinase receptors [6], surgical resection is still considered the treatment of choice for GIST [7].

We present short and long-term results of surgical resection of patients suffering from different types of GIST over a period of 17 years.

## Materials and methods

Between 1987 and 2004, 25 patients (15 male and 10 female, mean age of 69 ± 8 years, range 34–80) underwent surgical resection for different type of GIST. The tumors were located in the stomach, small bowel, large bowel and peritoneum in 10, 10, 2 and 3 patients respectively.

All patients' data, tumor characteristics, surgical procedure, intra and postoperative complications, as well as follow-up and survival data, was entered in a specifically designated computer data base and was analyzed retrospectively.

Histopathological examination of surgical specimens was carried out using standard hematoxylin and eosin staining as well as specific immunohistochemical techniques allowing the identification of tumor's grade (low, moderate, and high), size, cellular pattern, stromal background, stage and number of mitosis at high-power field (HPF).

Using the standard histological classifications [3], 9/25 (36%) tumors were considered malignant, 2/25 (8%) of uncertain behavior (border line) and 16/25 (64%) benign.

The mean tumor size was 9.4 cm (range 1.2 – 30 cm): <5 cm diameter in 14/25 (56%) cases, 5–10 cm in 5/25 (20%) and >10 cm in the remaining 6/25 cases (24%).

Mitotic count was low (<10/50 High-Power Field) in 68% (17/25) of the tumors and was high (>10/50 High-Power Field) in the remaining cases (32% 8/25).

Using the "risk of aggressive behavior" classification proposed by Fletcher *et al *[2] (Table 1) tumors were classified as very low (11/25, 44%), low (4/25, 16%), intermediate (6/25, 24%) and high-risk (4/25, 16%). In the last 12 patients c-KIT analysis have been performed and all but one have been found to be positive.

**Table 1:** Proposed approach for defining risk of aggressive behavior in GIST, by Fletcher [2]

	**Tumor Size (cm)**	**Mitotic count**
**Very low risk**	< 2 cm	< 5/50 HPF
**Low risk**	2–5 cm	< 5/50 HPF
**Intermediate risk**	< 5 cm	6–10/50 HPF
	5–10 cm	< 5/50 HPF
**High risk**	> 5 cm	> 5/50 HPF
	> 10 cm	any mitotic rate
	any size	> 10/50 HPF

Five out of 25 patients (20%) were affected by significant coexisting malignancy: mesocolic located tumor associated with lung cancer (one patient); adenocarcinoma of the stomach (three patients) and, finally, cancer of the left colon was associated to pelvic GIST (one patient). These patients, even if both tumors were resected, were excluded from the survival analysis.

Clinical follow-up recorded late postoperative complications, recurrence (local and distant) and actuarial survival based on Kaplan-Meier method.

## Results

All patients underwent surgical resection. Table 2 describes the surgical procedure performed in relation to the site of the tumor. Macroscopically complete resection was achieved in 22/25 (88%) cases and in all these cases GIST belonged to either the very low, low or intermediate risk classes.

**Table 2:** Surgical procedures for resection of GIST

**Site (n)**	**Procedure**	**Complete/incomplete**
Stomach (10)	Local resection = 6 (60%) Partial gastrectomy = 3 (30%) Local resection + excision of diaphragmatic tumor deposits = 1 (10%)	Macroscopically complete Macroscopically complete Incomplete
Small bowel (10)	Local resection = 4 (40%) Small bowel resection = 6 (60%)	Macroscopically complete Macroscopically complete
Peritoneum (3)	Local resection = 1 (33.3%) Bulky excision = 2 (66.7%)	Macroscopically complete Incomplete
Colon (2)	Local resection = 1 (50%) Left colon resection + pelvic metastasectomy = 1 (50%)	Macroscopically complete Incomplete

In 3/25 (12%) cases, radical resection was not possible due to diffuse disease (2 cases) or local infiltration (1 cases) and the final histopathology described the specimens as malignant, high-risk GIST (Table 1). There was no intra or postoperative mortality. In one case (5.2%) accidental intraoperative spleen damage occurred that was treated by splenorraphy.

Postoperative mortality rate was 32% (8/25). Four cases died of wound infection, two of lung atelectasis, one of pleural effusion and one of central venous catheter infection. The mean follow-up period was 54 ± 10 months (range 22–120).

**Figure 1:**
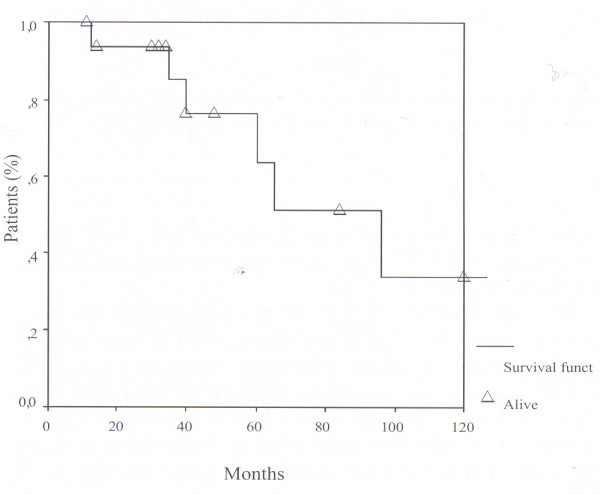
Kaplan Meier survival curves showing overall survival.

The 5 and 10-year overall actuarial survival using the Kaplan-Meier curve was 65% (standard error 21%) and 34% respectively (standard error 13%) with a mean survival of 85 ± 16 months (Figure 1).

**Figure 2:**
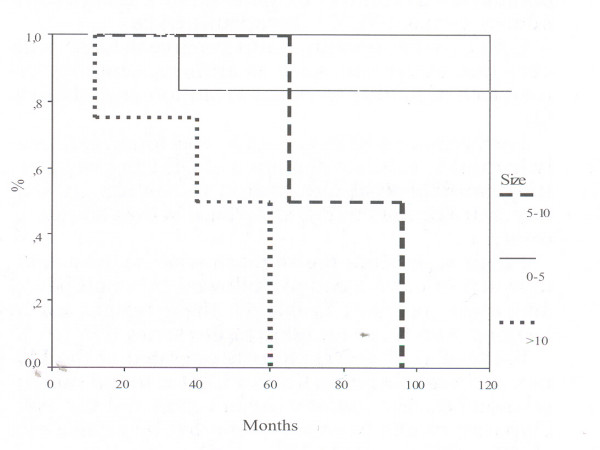
Kaplan Meier survival curves showing survival by size of the tumor.

The mean survival was 126 ± 23 months for tumors <5 cm, 81 ± 16 for tumors 5–10 cm and 43 ± 12 for tumors >10 cm (Figure 2). Difference between <5 cm and >10 cm was statistically significant (p < 0.05; log rank test).

**Figure 3:**
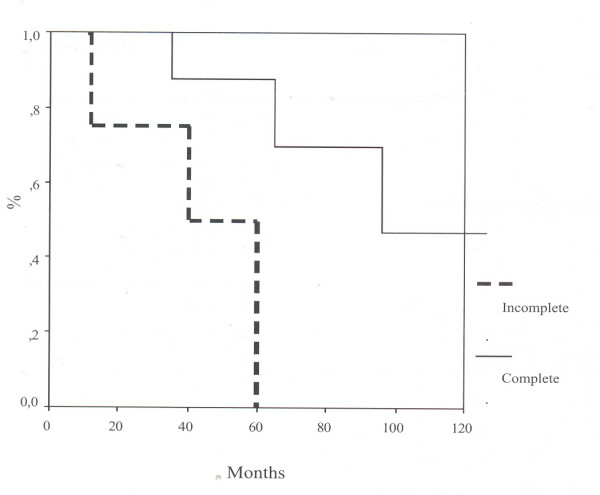
Kaplan Meier survival curves showing survival by completeness of excision.

Mean actuarial survival after complete and incomplete resection was 105 ± 19 versus 43 ± 12 months respectively (Figure 3) and this difference was statistically significant (p < 0.05, log rank test). High-risk tumors had a significantly shorter survival than low and very low risk tumors (mean 43 versus 144 months, p < 0.01). There was no statistically significant difference in survival by the location of tumors.

## Discussion

Gastrointestinal stromal tumors are rare neoplasm that may arise virtually in any part of the gastrointestinal tract. It has been recently proposed that GISTs may develop from the interstitial cells of Cajal [1], since the same immunohistochemical markers can identify GIST. Presence of CD34 antigen and the c-KIT proto-oncogene (CD117) represent the histological features of these tumors [1,3,8].

Other studies [1-3,8] also demonstrated the diagnostic role of c-KIT expression, now considered a highly specific marker for GISTs. c-KIT expression can also be used for medical therapy [2], since new drugs, (STI-571 imatinib), is characterized by a selective action on tyrosine kinase receptors, and it has been recently used with good results [6,12,13].

C-KIT always immunostains positively in GISTs, with very rare exceptions, due to artifacts, sampling errors, lack of kit caused by clonal evolution or mutations [2]. Furthermore c-KIT expression is extremely helpful to validate diagnosis of GIST in case of extra-gastrointestinal localization (omentum, mesentery, retroperitoneum or elsewhere in the abdominal cavity) [2].

In our experience the stomach was the most common site of GIST location, followed by small bowel and other locations (Table 1): these results are in keeping with those found in larger series [9].

Brennan *et al*, in 200 patients found no correlation between tumor's site of origin and survival. Opposite results have been reported by Lillemoe *et al *[10], in 133 resected GIST, where survival was, indeed in relationship with tumors' site.

There are difficulties to classify benign or malignant GIST using the standard criteria commonly used for other tumors and most authors agree that tumors size and the number of mitoses at HPF are the most important factors related to prognosis [2,3,5]. These findings allowed Fletcher *et al *[2], to propose a "risk of aggressive behavior" classification of GIST considering only size and HPF mitotic count (Table 1).

Surgical resection is still "the gold standard treatment" for GISTs, allowing to reach a cumulative 3–5 years survival of almost 50% and 35% respectively [1,3,4]; these findings are confirmed by our series (Figure 1).

Our study confirms that size of the tumors as well as mitotic count are highly related to the prognosis: patients suffering from GISTs less than 5 cm in diameter have a significantly longer survival than patients with bigger tumors: 126 ± 23 months for tumor <5 cm, 81 ± 16 for 5–10 cm and 43 ± 12 for >10 cm GIST (Chart 2) (p < 0.05).

Similar results have been reported by Brennan *et al*, [4] as well as other authors [7].

Using the "risk of aggressive behavior classification" (Table 1) [2] for our patients, we confirmed that low number of mitosis at HPF are related to prognosis: we found a significantly longer survival in very low and low risk group compared with high risk group (p < 0.05).

In our experience macroscopically complete resection was possible in 21/25 cases (84%) (Table 2); the presence of residual tumor was significantly related to early recurrence and short survival (Figure 3).

The negative effect of macroscopic residual tumor is well known: Ott *et al*, [7] and Brennan *et al*, [4] found a significantly longer 5 year survival rate when GIST were completely removed (42% versus 9%).

We found no difference in term of survival considering different tumor locations, but the prognostic significance of the site of origins in term of survival is still controversy. While Brennan *et al*'s [4], finding are in keeping with ours, results from the 164 patients treated at the Johns Hopkins Hospital showed longer survival for esophageal and duodenal located GIST [10].

Since approximately 50% of the patients will not survive more than 5 years even when complete resection is achieved, several chemo-radiotherapeutic adjuvant regimens have been proposed.

As these tumors mainly arise in gastrointestinal tract and irradiation may produce severe damage on adjacent organs, radiotherapy seems to be indicated only to prevent local recurrence in selected cases of rectal GISTs [7,11].

A combined European/Australian study [2,12] has been started, in order to test the effectiveness of a new chemotherapy regimen based on imatinib mesylate STI-571: a tyrosine kinase receptors inhibitor [6], highly selective on GISTs and able to perform a "target" therapy, based on the c-KIT expression (CD117) of these tumors. Preliminary results of the first phase trials demonstrated up to 80% of partial response in patients suffering from metastatic or recurrent GISTs treated by imatinib.

Similar results have been obtained in a phase II and III study, started in the United States, on unresectable or metastatic GISTs [2,13].

An adjuvant or palliative therapy by oral administration of imatinib is now considered mandatory for unresectable or metastatic diseases as suggested by a recent and extensive review by Bucher *et al *[14], while its role in potentially resectable GITS or as neo-adjuvant regimes has to be demonstrated yet.

## Conclusion

Our experience confirms that GISTs are uncommon and aggressive cancers. The prognosis is strictly related to size and number of mitosis at HPF. Complete surgical resection of the tumors still remains the only chance of long term survival for these patients. Imatinib therapy should be used for patients not suitable for surgery due to poor general condition or once a complete resection it is not technically possible. Its role in adjuvant settings after complete excision is still controversial.

## Competing interests

The author(s) declare that they have no competing interests.

## Authors' contributions

**LB**: performed the literature review and contribute to manuscript writing

**AB**: supervised the manuscript preparation

**GD**: helped during the manuscript preparation (introduction and discussion) and literature review

**FR**: helped during the manuscript preparation especially in the statistical methods and discussion

**RD**: helped during the manuscript preparation and final revision, performed most of the surgical procedure
